# Examining the Process of Modal Choice for Everyday Travel Among Older People

**DOI:** 10.3390/ijerph17030691

**Published:** 2020-01-21

**Authors:** Jean Ryan

**Affiliations:** 1 Division of Transport and Roads, Department of Technology and Society, Lund University, P.O. Box 118, 22100 Lund, Sweden; jean.ryan@tft.lth.se; 2 K2, The Swedish Knowledge Centre for Public Transport, Bruksgatan 8, 22236 Lund, Sweden; 3 Centre for Ageing and Supportive Environments, Department of Health Sciences, Lund University, P.O. Box 157, 22100 Lund, Sweden

**Keywords:** modal choice, modal options, older people, mobility, capability, Sweden

## Abstract

Modal choice is a prominent concept within transport studies. However, the term is often used quite loosely, with little known about the factors lying behind the choice, the alternatives available to a person, and whether the person had a ‘choice’ to begin with. This study draws on a travel survey among older people living in Sweden’s large metropolitan regions. The questions posed as part of this survey facilitate a greater insight into the processes at play behind modal choice. An analysis of the differences between: (1) the range of modal options available to respondents and (2) the modes selected from this range (modal choice) is presented. An analysis of the respondents’ reasoning for choosing the modes they did and not the others they could have chosen is also presented. It was found that more than a quarter of respondents have the option to use and actually use all modes for everyday travel. The car is more inclined to be selected among those who have a range of different modal options. Suitability and comfort are the two main reasons given for modal choice. More positive reasons are given for actively selecting walking and cycling, whereas the motives behind the selection of the car instead tend to be framed as reasons for not selecting other modes. Adaptive preference and adjustment effects are also apparent in the selection processes. This study gives us a deeper understanding of the intricate mechanisms and reasoning at play behind the process of modal choice among this group. In this way, we have a better basis for shaping and implementing measures to promote and encourage sustainable mobility, in such a way that the well-being of older people is also supported.

## 1. Introduction

### 1.1. The Links between Ageing, Potential Mobility, Choice and Well-Being

The importance of having the possibility to participate in society while ageing has been studied and documented in several different contexts [1,2,3,4], as has the intrinsic value of mobility in itself [1]. Having the possibility to participate in various out-of-home activities has been found to be central for well-being outcomes during the later stages of life [5,6]. This makes exploring the potential means of getting to these activities a key societal concern.

Having a range of alternatives from which to choose is widely considered to be beneficial for a person’s well-being ([7], pp. 33–39). In terms of daily mobility, this can relate to the range of everyday activities in which a person can participate [8,9]; the time frames during which a person can participate in said activities [10,11]; and the mode(s) of transport a person can use to reach said activities [12]. The importance of having a range of modal options from which to choose (referred to as ‘robustness’), and indeed option value, have been emphasized as important elements of accessibility [13]. This robustness is considered key as it means that an individual is not reliant on a specific mode (with its inherent limitations) but instead has the option to be flexible and free to choose among several modes. This has also been discussed as a multimodal advantage, with those limited to just one mode said to be subject to a corresponding monomodal disadvantage [12].

The potential for mobility, most often in the form of accessibility (as opposed to mobility itself), is becoming a more prominent focus [14]. This potential has in turn been found to have strong associations with freedom and autonomy, particularly for older people during the ageing process [15]. This potential is also said to comprise important elements of well-being in itself [7]. Accessibility, motility and capability are key concepts which in recent years have been gaining ground as part of discussions surrounding potential mobility see e.g., [16,17,18] for elaborations of each concept. Instead of how, when, where or with whom a person travels, their possibilities or opportunities to do so are becoming the focus within research.

### 1.2. The Capability Approach as a Conceptual Framework

The Capability Approach (CA) has been receiving increasing attention as a conceptual framework in transport research [19,20] not least due to its focus on the size of the choice set and how it is tightly linked to equity concerns [21]. The CA has an emphasis on realizing potential and thus a focus on Eudemonic as opposed to Hedonic aspects of well-being, or becoming, being or living one’s ‘true self’ [22]. The CA emphasizes the direct links between greater freedom—conceptualized as a larger set of ‘capabilities’—and well-being [23]. Well-being, in turn, is influenced by the capabilities which are operationalized as functionings or the ‘beings’ and ‘doings’ of the individual [23].With respect to daily travel, this is related to the pre-conditions for a person’s mobility; the options, capabilities or scope a person has with respect to mobility. The ties between this scope for action and realized mobility is framed as *evaluation* and *selection* of the best alternative, as deemed ‘best’ by the individual herself.

Choice is a central concept in the field of transport and mobility, with choice—particularly modal choice—a key aspect of many studies e.g., [24,25]. Revealed behavior (‘functionings’ within the CA) is the main consideration for many studies within transport. However, revealed behavior only tells us a very limited amount about a person’s range of options [7] (pp. 36–37). If we are to focus on choice, the *range* of options—and the individual’s selection from this range—is a more appropriate realm of analysis [7].

### 1.3. Ageing and a Reduced Scope for Action

A person’s choice set with respect to mobility is largely considered to reduce in size in tandem with the ageing process [26,27], often as a result of declining health, reduced cognitive or physical capacity [28,29], changes in household composition (for instance, the death of a spouse) [30], or changes in personal financial circumstances [31]. For example, a person’s scope for action may decrease in terms of the cognitive or physical capacity required to negotiate the environment(s) in which she moves, or a person’s social capital might be diminished if her social network contracts [27,32,33]. However, the scope for action may also increase in some aspects of life as people age. For instance, as one retires, she may enjoy a greater level of flexibility in terms of time [34]. Some have noted that for older women in particular, reliance on a partner for transportation can ultimately result in a decreased scope for action [8,10]. At the same time, caring for a person within one’s social network, even just keeping them company, can also mean a reduced scope for action [30]. Not having access to technology, or not having the possibility to use it can, on the other hand, result in reduced scope for action, particularly as we witness the digitalization of many forms of transport. This can again affect women in particular [35,36].

Maintaining the modal choice set in as far as possible could be highly beneficial for older people, in particular older people who have a more limited range of options from which to choose in comparison to what they may have had during earlier stages of life. It has been found that modal options are important in terms of facilitating the fulfilment of travel needs and activity wishes, while a lack thereof can have the opposite effect [37,38]. A link has even been found between the use of a narrower range of modes and loneliness [39], with another study finding that some modes of transport are more closely associated with a greater scope for action than others [11]. It has even been emphasized that having options to choose from might not appear to be so important during the earlier stages of the ageing process but its importance may become more apparent during the later stages of the ageing process, if or when one has a reduced scope for action and more difficulty participating in everyday out-of-home activities [40]. While several studies have highlighted the importance of the car as a modal option during later life [41,42], the straightforwardness of the relationship between the use of the car and the fulfilment of travel needs has also been questioned, particularly when one considers and accounts for different socio-demographic factors [43].

While potential mobility (encompassing modal options) and realized mobility (encompassing the mode(s) chosen) among older people have been explored in previous studies e.g., [37,38,39], investigating the process from potential to realization and the different kinds of motivation and reasoning behind the selection of some options over others is less explored. Furthermore, if we are to treat choice as an element of well-being, it is important to analyze the process of choice, and to distinguish between actually having chosen something and doing the same thing without having had a choice. Many empirical studies focusing on modal choice, particularly those based on standard travel survey data, do not make such a distinction.

This study examines the process from potential to realized mobility among older people, with a specific focus on modal choice. The differences between the range of self-reported modal option(s), the modal option(s) selected from this range, and the reasons behind the selection of some modal options over others are explored. Links and exchanges between the experienced so-called ‘real’ options and realized behavior are then analyzed. In this way, we gain a greater insight into the different dynamic elements at play in the process from potential to realized mobility (evaluation as part of the CA), and the different forms of reasoning associated with the selection of different kinds of options.

## 2. Materials and Methods

This study draws on a capability-based travel survey among older people (aged 65–79) and living in Sweden’s large metropolitan regions; Stockholm, Gothenburg and Malmö. This meant that the survey was purposefully designed and shaped to capture elements of the CA. People within this age group (65–79) tend to have a greater scope for action than those aged 80 and above [44]. The questionnaire was developed with the intention of capturing three specific elements of the CA: resources (e.g., income, social capital, access to a bicycle, holding a driving license, etc.), capabilities (the range of different possibilities possessed by a person to achieve ‘beings’ and ‘doings’ of value) and functionings (the selected ‘beings’ and ‘doings’ of value). For a more detailed description of what the different elements of the CA entail, see [23]. The questionnaire and the Computer-Assisted Telephone Interviewing (CATI) script was formulated, tested and adapted a number of times before interviewing began. Ethical approval for this study was granted by the Regional Ethical Approval Board in Lund, Sweden in September 2015 (Dnr 2015/447).

Sweden’s three large metropolitan regions (LMRs), Stockholm, Gothenburg and Malmö, comprised the study area. These three LMRs are geographically delimited based on commuter patterns and movement between the central municipality and outer municipalities [45]. For this study, it was considered that LMRs would include and approximately reflect the relevant geographical areas of respondents’ everyday activities.

A stratified random sample of 3200 people aged 65–79 and living in Sweden’s LMRs was drawn from a population register, with contact details containing a combination of landline and mobile phone numbers. Eleven people opted out before data collection began. A further two people opted out while data collection was underway. Interviewing was conducted by a small group of trained interviewers at a well-established and ISO-certified market research company. The interviews were carried out using CATI, allowing for more interviewer-respondent interaction so as to keep misunderstandings to a minimum and to facilitate a two-way communication process, when compared to other methods of data collection (e.g., compared to a postal survey or web-based survey).

The interviews were conducted over the course of a three-week period during November-December 2015. The progression of the data collection was followed in order to gauge whether respondents understood and answered the questions in a consistent manner. From the remaining sample, attempts were made to contact 2119 people. These people were randomly selected but the probability of being selected changed successively with the completion of each additional interview. From those contacted, 1150 agreed to participate in the study, resulting in a response rate of 54% (56% for Stockholm; 52% for Gothenburg; and 55% for Malmö). In total, 1149 valid interviews were conducted (*n* = 383 in each LMR). The reasons given for not participating were (1) the person has a principle of not participating in surveys (29%); (2) the person did not answer (the phone) when they were contacted (18%); and (3) the person did not have time to participate when contacted (13%). All interviews were carried out in Swedish and over the phone. This meant that some people who were contacted could not participate. Among those who could not participate, 5.7% had difficulty speaking, spoke unclearly or did not speak Swedish. See [46] for a more detailed description of the sample, questionnaire and descriptive statistics.

For this study, the focus lay on the process from potential to realized mobility among older people, with a specific focus on modal choice, and the reasoning behind the selection (and use) of specific modes over others. This meant that the elements of the material related to ‘capabilities’ and ‘functionings’ were the main focus. The analysis of this material was divided into the following parts:

a) A descriptive analysis of the differences between the range of modal options available to respondents and the modes then selected from this range (modal choice).
b) A descriptive and explorative analysis of the reasons behind the selection (and use) of said modes and the links between such reasoning and the different forms of selection processes.

As part of the survey, respondents were asked to report which modal options they have the possibility to use for their everyday travel within the local region, even if they never use them. For this analysis, driving a car, using public transport (PT), walking and cycling were included as modal options. This material formed the basis for the ‘scope for action’, or the range of options from which individuals could choose. It is important to note that whether or not the individual considers it possible to use different modes of transport is highly subjective and is purely based on the perceptions of the individual. As such, two people who, for instance, appear to have all the same pre-conditions to cycle (e.g., access to a bicycle, physical capacity, short distances to activities of interest) may report different possibilities to cycle. This could be considered problematic in the sense that comparisons between individuals are not straightforward. However, the individual’s perception is the key aspect of interest here, as it reflects the situation as perceived by the individual and thus, her perceived capability with respect to mobility.

The respondents were then asked to state which options from this range they actually use, indicating ‘realized mobility’ and ‘modal choice’. Here, although the respondent may have reported having a range of modal options from which to choose, she might perceive that she does not actually have a choice with respect to the mode she then uses. Similarly, the term choice may be perceived differently by respondents, with some perhaps reporting that they have the freedom to choose, and others reporting that they do not, despite being subject to the exact same circumstances. Respondents were then asked to give some reasons for why they opt to use these specific modal options and not the other modal options they could have selected. These reasons were processed in part as pre-codes. These were reasons, based on literature review, that were predicted to be given by respondents in advance of the survey being conducted. Respondents could also give open-ended reasons or accounts for their selection. It was possible to give several different reasons for selection, both in the form of pre-codes and open-ended answers. This meant that those who stated that in some way one mode was ‘more suitable’ than another often expanded upon their reasoning in the form of an open-ended answer. The actual formulation used in the open-ended answers was analyzed in a qualitative manner, while the information provided was subsequently coded into usually 2–3 different codes. Many respondents gave a combination of several pre-codes as well as an open-ended explanation. The prevalence of the different kinds of codes given by respondents were analyzed and subsequently combined into themes. The unique combinations of different codes provided were also analyzed, as was the actual wording given by respondents (in the open-ended element) for their modal choice. This material formed the basis for the analysis of the motivation and reasoning behind different forms of modal choice.

The approach and analysis for this study can be regarded as a combination of quantitative and qualitative methods. The material obtained through the survey allowed for comprehensive, broader, descriptive analyses. However, such analyses are somewhat superficial in nature in terms of the actual meaning for respondents. The subsequent, more qualitative analyses allowed for a deeper and more detailed insight into the nuances behind the respondents’ reasoning, allowing us to a gain a clearer understanding of the processes at play.

## 3. Results

Below is an account of the results for each part of the analysis.

### 3.1. The Differences between Scope for Action and Modal Choice

**Figure 1:**
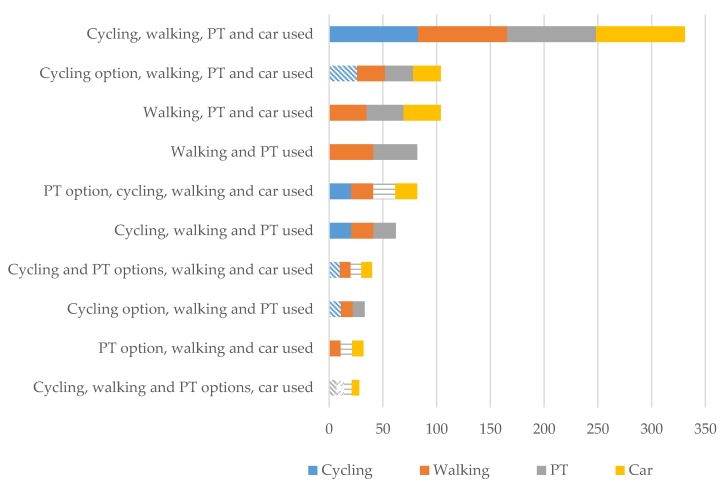
Illustration of the most common modal choice processes. Those filled with a pattern indicate a modal option that has not been used. The modes encompassed in each selection process are given equal representation in the chart. This, however, is purely for illustration purposes and is not intended to reflect the extent to which individuals use the respective modes; PT—indicates public transport.

An outline of the various combinations of modal options, choice (use) and the frequency of the different choice processes among respondents is presented in Table 1, with the most common selection processes illustrated in Figure 1. This gives us an insight into the scope for action and the selection of different modal options among respondents.

More than one-quarter of respondents (28.8%) belong to the same category; having and using all four modal options. This category can be considered to comprise those at a relative multimodal advantage to the remainder of respondents [12].

By looking at the selection processes taking place among the second and third largest categories, it is clear to see that cycling is the most ‘sensitive’ with respect to selection; it being the first option to drop off among all the modal options. Those cycling, walking and using the car, yet choosing not to use public transport despite it being regarded as a modal option is relatively large (7.1% of respondents).

Those who consider that they have walking, cycling, public transport and the car as modal options, yet selecting to use just the car is a larger group than expected, although still relatively small at 2.4%. Those with walking and cycling alongside the car as options yet choosing the car comprise a further 1.4%, with those with public transport as an option yet choosing the car making up a further 1%. Those who have the possibility to cycle yet choose the car comprises a further 0.5%, while those with both cycling and public transport as options yet choosing the car makes up another 0.3%. This means that the group actively selecting the car among a range of modal options amounts to 5.6% of respondents. The proportions of respondents actively selecting the other modal options among their respective ranges are much smaller, with the combinations of their respective categories largely only making up a fraction of the combined category actively selecting the car.

Not all respondents have options, with 1.0% of respondents stating that they have no modal options whatsoever (or not answering the question). Several respondents state that they have just one or two options, and yet do not use those one or two options. Others state that they have just one option and use that one option.

Some of the socio-demographic differences in modal choice processes are presented in Table 2. The variables of income (‘lower income’ encompasses a monthly household income of up to 16,666 Swedish crowns (SEK)), gender (male and female) and household status (cohabiting or not) were included in the analysis. Table 2 indicates the key differences (and similarities) between socio-demographic groups, although no detailed analysis of statistically significant differences has been carried out. Walking and using public transport is spread evenly between income groups, while women are and those not cohabiting are somewhat overrepresented for this modal selection process.

While having cycling as an option, yet choosing to walk and use public transport was linked to an overrepresentation of women, suggesting that the sensitivity of cycling for modal selection may be gendered. Actively selecting to use the car despite having other options is associated with an overrepresentation of men, suggesting that this too may be gendered. Those with lower incomes appear to be overrepresented in the use of cycling, walking and public transport as modes of transport (as a unique combination) while those cohabiting appear to be more inclined to actively select the car. However, this is just intended to give a brief overview of the sample. Further, more detailed statistical analyses are required in order to draw any robust conclusions regarding the differences outlined.

### 3.2. The Reasons Given for Modal Selection

An outline of the different kinds of reasons given by respondents for their modal choice is presented in Table 3.

The most common reason given by respondents for choosing the mode(s) they use over the mode(s) they could have used was that they are *more suitable*. Almost one-third (30.6%) of those who answered this question gave this as their one and only reason for choosing the mode(s) they did, while this reason accounted for 36.3% of mentions, either as a sole reason or combined with other reasons. When combined with other reasons or open-ended answers, ‘more suitable’ appears to be related to matters of convenience, and the mode fitting the purpose intended, be that using public transport in order to avoid difficulty getting parking, or opting to walk because activities lie within walking distance. The explanation attached to the pre-code *more suitable* meant that both positive and negative reasons could be coded in this way i.e., choosing a mode due to it being more efficient in terms of time or *not* choosing another mode because it does not suit the purpose of the trip. The second most common sole reason given was this/these mode(s) being *more comfortable* than the one(s) not chosen (at 15.2%). This reason accounted for 23.1% of the total mentions. Several respondents also mentioned a combination of just these two reasons (more suitable and more comfortable) (7.9%).

A rather small proportion of mentions (1.5%) related to choosing mode(s) because they are *better for the environment/for environmental reasons*. However this was usually combined with at least one other reason, and rarely given as the only or main reason, often combined with *enjoyable* or *exercise/wants to move as much as possible*. At the same time, *enjoyable*, *less expensive* and *better for the environment/environmental reasons* also commonly featured as a combined reason for modal selection. These combinations of reasons were often linked to the selection of more active modes (walking and cycling) when the open-ended statements were analyzed.

Furthermore, people mainly mentioned exercise as a reason when choosing to walk or cycle. However, this was also given as a reason for choosing to use public transport, with public transport trips sometimes described as comprising an element of exercise. Exercise when walking to/from public transport was even given as a reason for choosing one form of public transport over another e.g., opting for a longer walk to the train station instead of walking to a nearby bus stop.

Fewer than expected reported *Health reasons/poor health/no energy* as their sole reason for modal selection (3.2% of respondents, 3.6% of mentions in combination with other options). Having poor health was often combined with another reason and was rather rarely given as a reason on its own.

There was, in fact, another more positive relationship with health linked to modal selection, with 1.4% of mentions related to exercise, with several stating that they make this specific selection as exercising is healthy.

A rather small proportion (2.2%) mentioned *faster/speed* (being faster than other modes) as their only reason for choosing the mode(s) they do over the modes they do not, with the proportion of mentions accounting for a more considerable 6.4% when combinations with other reasons were included. Being *less expensive/financial reasons* was also mentioned by surprisingly few (3.2% of reasons mentioned). Interestingly, these two reasons (*faster/speed* and *less expensive/financial reasons*) were often given in combination with one another. When focusing on the open-ended reasons, being ‘inexpensive’ often meant that a person had, for example, already paid for the car and did not want the extra expense of paying for public transport, or indeed, that a person had already bought a monthly or yearly public transport ticket and wanted to make the most of it. This was also related to choosing to walk and cycle because these modes do not cost anything, in the eyes of some respondents. There was only one mention of free public transport and this was by a person living or travelling in Gothenburg.

*Proximity* as a sole reason for modal selection represented a larger proportion of respondents than expected (2.4%, 3.0% of total mentions), with 1.0% mentioning a combination of suitability and proximity for their modal selection.

The reason *flexible, depending on the situation*, using all options or the strategic use of modal options accounted for 1.7% of sole mentions (1.3% of all mentions), while 2.3% of respondents mentioned having no choice/not much choice or having no alternative(s) as their sole reason for choosing the mode(s) they do. A small proportion (1.9%) explicitly stated that they have no need for alternatives, and gave this as their only reason for modal selection. While 2.4% stated that the sole reason behind their use of these modal options was simply that these were the options available to them. Some of the open-ended answers alluded to this being the life (style) that was chosen; their modal options were limited, but this was a conscious decision. All of the reasons encompassed in these codes allude to having a limited choice set. However, interestingly, the individuals’ perceptions of their limited choice set differ considerably, with some expressing implicit dissatisfaction (*no choice/not much choice or having no alternative(s)*) and others expressing implicit satisfaction (*no need for alternatives*) despite perhaps having the same limited range of modal options. Again, the highly subjective nature of the survey means that individuals’ perceptions of what is/is not an option could vary to a considerable extent. Some respondents may report not having a choice with respect to which modes they use despite considering that they do have other modal options. Here, there are two aspects to consider, the differences in the scope for action among respondents, as well as the differences in the perceptions of their scope for action.

Respondents were inclined to give more positive reasons for selection when selecting more ‘sustainable’ modes. There was a sense of ‘positive’ or ‘active’ selection of the more active modes, with the environment, health, exercise and enjoyment linked to the selection of active or more sustainable modes. Several who chose walking and cycling explicitly mentioned proximity and accessibility to activities, mentioning that they live ‘strategically’ or ‘centrally’ and do not have any need to use a car when everything—or most activities—are within walking or cycling distance of their residential location. This is in contrast to the selection of the car, with this selection mechanism characterized as more passive, as reasons for not selecting other options, as opposed to direct reasons for selecting the car. Several talked about there being no alternatives; this was their option, and being locked into a lifestyle with this modal option. More than expected talked about complications related to the car i.e., having to find and pay for parking being the most prominent (0.3% of mentions). Fewer than expected mentioned freedom or wanting to have flexibility as a reason for choosing the car. Although a few open-ended responses did imply these aspects:

*“I come and go without a timetable.”*

Others took the car as a given or had normalized the use of the car as a sort of ‘de facto’ option, stating that they would only look into other options if it was not possible to use the car. Others questioned why one would even think about using other options when then the car is the only necessary option:

*“I use my own car as the first choice.”*

Some mentioned that their lifestyle dictates which mode(s) they use, meaning that having to carry heavy loads or living considerable distances from activities in remote locations result in the car being the only viable option. Fewer referred to not choosing public transport but when they did they talked about the non-existence of public transport, or mentioned that the destinations that could be reached using public transport were very limited, or that the public transport itself (i.e., the bus stop) was located too far away.

Reliability was mentioned by fewer than expected and was usually given in combination with at least one or two other reasons. In the literature, reliability is often mentioned as an important aspect with respect to modal choice [37,47] but as a theme, it was less apparent here.

There were rather clear adaptive preference effects see [48] (pp. 83–84) at play with some people mentioning that they should not expect to be able to use other modes as they are sick or have poor or declining health, or mentioning that they have no choice, and that the only options that exist for them are the ones they use. Indications of adjustment related to the ageing process was also present. This materialized in the form of respondents discussing having stopped using certain modes, having had a fall, or having begun to experience declining health:

*“I am a pensioner. There is no need for me to travel every day.”*

The use of the tram became apparent as a very prominent mode of transport in Gothenburg, as several respondents living in this region went out of their way to mention their specific use of the tram, with habit an intertwined theme with the selection of this mode. Mentions of the tram in Gothenburg were much more evident than any other form of transport in any other area:

*“ Because the tram goes exactly where I want it to go, and very often. It suits me fine. The tram is the best thing that exists.”*

The use of the tram seemed to be associated with a form of pride or a deeper association with the central parts of Gothenburg. In this sense, some of the reasons given suggested that the identity of the city seemed to be connected to the identity of the person, which in turn points to the symbolism of the tram as a mode of transport, and indeed its social meaning as a mode of transport [1,49,50]. Walking and the use of the tram were often discussed in tandem, alluding to the complementary nature of the two modes.

Several mentioned the needs or wants of another person (most often a spouse) influencing their modal choice, e.g., having to give the spouse lifts, or not being able to use certain modes because they usually travel with the spouse. On the other hand, others talked about choosing the car because they had to give grandchildren lifts and had installed car seats for this purpose. Others talked about not being able to use the car and instead using public transport when travelling with grandchildren, or using public transport to reach their own children’s homes to visit them and grandchildren.

A number of respondents (1.3% of mentions) discussed tailoring their choice according to their needs. This meant having several options but, for instance, opting to use public transport in the city center or cycling or walking for shorter distances, but when it came to longer distances, using the car or public transport On the other hand, others (rather few) mentioned using the car for shorter distances, simply because they were used to using the car.

### 3.3. The Links Between Modal Selection Processes and the Reasons Given for Modal Selection

The codes presented in Table 3 were combined to form overarching themes, as presented in Table 4.

Only the first recorded reason given by the respondent was used to form the codes, and overarching themes. This was because many respondents gave a range of different reasons (some gave up to eight reasons) that could not be placed into just one coding category or theme. This was in order to limit and simplify the categories produced by the cross-tabulation with the selection processes, presented in Table 5, and is, unfortunately, a limitation of this part of the analysis.

The cross-tabulation of the different codes (reasons for modal selection) with the selection processes is presented in Table 5. Just some categories of modal selection processes were included, again in order to limit the number of categories produced. This also meant limiting the analysis to a number of more interesting groups, or rather, those who had actively selected a specific mode (or modes) when they had the possibility to select other modes. It is also important to note that many of the groups and sub-groups are very small so it is not possible to draw any robust conclusions from these small numbers. They are instead intended to give an illustration of how the respondents answered and reasoned.

Those who actively chose the car were also more inclined to give extra benefits as a reason for choosing the car, although in most if not all of these specific cases, respondents were referring to enjoyment and freedom, and not exercise or the environment. As detailed in Section 3.2, those giving these reasons generally gave them in combination with other reasons, and were most often not the first reason recorded, and therefore not presented in Table 5.

## 4. Discussion

The aim of this study was to examine and gain a greater insight into the process of selection from potential to realized mobility among older people aged 65–79, with a specific focus on modal choice. The differences between the range of modal option(s), the modal option(s) selected from this range, and the reasons behind the selection of some modal options over others were explored.

One of the main findings was that a sizeable proportion (more than a quarter) of respondents have the option to use and actually use all modes for everyday travel. Although this can be considered a rather positive finding, this group can be considered to comprise those at a multimodal advantage relative to the remainder of respondents, a remainder which in turn comprises the majority.

If we are to consider that a greater range of modal options can imply a greater freedom with its inherent links to well-being [7], then this group certainly fares best, especially when taking the findings from other studies into account [37,38,39]. However, others might have fewer modal options but still feel that their activity needs are met (e.g., those who expressed having no need for more alternatives). Here, it is difficult to decipher whether this is an indication of adaptive preference, or if this group in fact does have all of their mobility needs covered with just one option. Nonetheless, having just one or very few options might imply being at risk later on, if said modal options are no longer viable in later stages of life.

Cycling as a modal option appeared to be the most sensitive with respect to selection; it being the first option to drop off among all the modal options. This is in line with previous findings where cycling has been found to be quite difficult for older people, with reports of issues with traffic safety, concerns with cycling environments and the behavior of other road users [49,50]. This sensitivity appeared to be gendered. Although further analysis is required to confirm this. At the same time, other studies have pointed to the benefits of cycling for older people [51,52]. Here, just like with driving cessation, cycling cessation (no longer having cycling as a modal option, and thus experiencing a reduced scope for action) requires adjustment and reconfiguration of mobility, and in some cases, participation in society [50]. However, for this study, we do not know whether the exclusion of cycling from the selected modal options is due to the individual having ceased cycling, or simply never having cycled.

There were groups who perceived that they had no choice and just used the one or two options available to them. This could either indicate a difference in modal options, or just a difference in perceptions of modal options and choice among respondents, or indeed, both. This is an important aspect and limitation of subjective material and a focus on perceptions. Similarly, there were very different perspectives on having a limited choice set, with some respondents expressing implicit dissatisfaction (*no choice/not much choice or having no alternative(s))* and others expressing implicit satisfaction *(no need for alternatives)* despite perhaps having the same limited range of modal options. This finding calls into question the conceptualization of modal choice in empirical studies and underscores the importance of exploring the notions of choice and how choice is framed and deciphered as part of empirical studies. Moreover, there may be differences between an observer’s account of the individual’s choice and the individual’s account of her choice, which can also be somewhat problematic, particularly when attempting to objectively assess differences among groups and between people [53]. Here it is rather difficult to decipher wherein the difference lies—a difficulty which has been highlighted by several studies (see [53] for an overview). In this sense, engaging different methods and different perspectives (both ‘objective’ and ‘subjective’) might assist in giving a more comprehensive and accurate picture of choice. Although we can question whether an option can really be considered an option if it is not the individual’s perception that this is the case.

Suitability and comfort were the two main reasons given for modal choice. There was a sense of ‘positive’ and ‘active’ selection of the more active modes, with more positive reasons such as the environment, health, exercise and enjoyment linked to the selection of active or more sustainable modes such as walking and cycling. This is in contrast to the selection of the car, with this selection mechanism characterized as more passive; as reasons for not selecting other options, as opposed to direct reasons for selecting the car were often given.

Nonetheless, the car was more inclined to be selected among those who have a range of different modal options, with poor health one of the main reasons given for selecting the car over public transport. This finding is in line with those of previous studies, where it has been found that health issues related to ageing are associated with more negative effects for public transport use than for the use of the car [8].

The selection of the car in this manner might suggest that the car is a *de facto* option for some—as alluded to in some of the reasons given by respondents. It also links back to the monomodality question, and the normalization of the car as a mode of transport, and how this reliance on the car can be problematic if driving cessation becomes a reality. In such cases, well-being can be significantly compromised, particularly if the cessation is involuntary [41,42,43]. In this sense, linking back to previous research, we can question how the car is often framed as the transport mode of choice [54,55], with this study revealing some of the more intricate elements behind this ‘choice’. This implies that choice might not be as clear-cut as it seems, particularly when considering the links between choice, scope for action, freedom and well-being.

The adjustment of expectations and preferences in accordance with disadvantage (often referred to as ‘adaptive preference’) [48] was evident in some parts of the empirical material. Such effects were apparent in the selection processes, with several respondents expressing that they know they have few or no options yet they do not feel that they should expect to have such a wide range of options. In some cases, this was in tandem with some clear adjustment effects, where respondents discussed having adjusted their expectations and actions as they aged. This also materialized in the form of respondents discussing having stopped using certain modes, having had a fall, or having begun to experience declining health [56].

This study facilitated a greater insight into the process behind modal choice; an insight which is often missing from discussions regarding modal choice. This study allows us to better understand the intricate processes at play and the underlying perceptions of individuals when considering their modal options and modal choice.

Linking back to the CA, we can see that there are indeed differences in the ways in which people perceive their options (their capabilities), and in how they evaluate their options and select and operationalize their capabilities (functionings). This is an inherent difficulty with the empirical application of the CA as it means that there are discrepancies when attempting to carry out inter-personal analyses, especially with respect to equity concerns. Nonetheless, the empirical material engaged as part of this study simultaneously allowed for both a broader and more in-depth analysis of the perceptions of individuals with respect to modal options and modal choice.

This study did, however, have some limitations. Its cross-sectional perspective did not allow for changes to be traced according to the ageing process. A longitudinal perspective would have aided us in understanding the selection process from an ageing standpoint. No ‘objective’ indicator was included, which would have allowed for a calibration of the self-reported responses, and would have given us a more nuanced perspective on some reports. This study did not explicitly include a geographical perspective. A lack of focus on geographical aspects may have resulted in some important considerations being masked as part of this study. Moreover, no comprehensive or clear-cut measure of subjective well-being was included in the study. This would have allowed for an empirical analysis of the links between modal options, modal choice and subjective well-being. This, in turn, would have facilitated a tighter connection to the CA and its conceptualization of the links between the size of the capability set and well-being. Nevertheless, this study did give a greater insight into the selection and reasoning processes behind modal choice, through the lens of those aged 65–79 and living in Sweden’s LMRs.

## 5. Conclusions

Through engaging the careful treatment of the process of choice and drawing on information about individuals’ reasoning with respect to the choice process, this study gave us a greater insight into the dynamic aspects at play with respect to modal choice for this group. Findings indicated that certain types of reasoning are related to the selection of specific modal options. Drawing on these types of reasoning and tailoring policy accordingly could prove fruitful in encouraging more sustainable travel behavior, and indeed promoting multimodality among this group. This would require a holistic approach by policymakers, encompassing not just transport policy but also community supports where the possibility to use other modes of transport (other than the car) are highlighted and promoted. For instance, training programs for using public transport could be employed. Solutions in the form of promoting multimodal trips (walking and public transport), highlighting the benefits in terms of exercise, experience and the environment could also be developed. In this way, individualized mobility suggestions could be developed so that people do not just use the car out of habit, and instead consider other modes. Not only ‘carrot’ but also ‘stick’ measures could prove effective. Several of the respondents remarked that they actively choose not to use the car because it is difficult to find parking. While others mentioned that they use public transport because they have a monthly ticket and want to make the most of it. Reducing parking opportunities alongside more attractive monthly pricing mechanisms could be another effective means of changing perspectives on modal options and intervening in the selection process.

Those who had several options were more inclined to choose to use the car. This is despite car use often being framed as a lack of choice. Importantly, ensuring that the car does not reign as the ‘automatic’ option is key to achieving a modal shift. For instance, making public transport and other modes more usable for people with health problems—through infrastructure, but also increasing awareness of other passengers and drivers—could be a crucial way of establishing such a change.

This study showed that the size and form of the choice set is important, and that it does matter whether someone chose from a range of options or instead just had to go with their one and only option. These findings have important implications for the consideration and treatment of modal options and modal choice within research going forward, presenting compelling reasons for including more detailed analyses of the processes behind modal choice. This approach could also be taken further by linking it to measures of subjective well-being, in order to draw clearer links between the scope for action (choice set) and well-being. Here, the focus was on those in the earlier stages of later life. These considerations could become even more tangible during the later stages of the ageing process.

By studying and plotting out the process of modal choice, the reasons behind the selection of specific modal options and links between these and other aspects of life, we have a better basis for understanding and planning for older people’s mobility. In this way, measures to promote and encourage sustainable mobility can be shaped and implemented, in such a way that the well-being of older people is also supported.

## Figures and Tables

**Table 1:** Frequency of the different modal choice processes.

Description of Selection Process	Frequency	Percentage
Cycling, walking, PT and car used	331	28.8%
Cycling option, walking, PT and car used	104	9.1%
Walking, PT and car used	104	9.1%
Walking and PT used	82	7.1%
PT option, cycling, walking and car used	82	7.1%
Cycling, walking and PT used	62	5.4%
Cycling and PT options, walking and car used	40	3.5%
Cycling option, walking and PT used	33	2.9%
PT option, walking and car used	32	2.8%
Cycling, walking and PT options, car used	28	2.4%
Car option, cycling and PT used	17	1.5%
Cycling option, PT and car used	17	1.5%
Cycling and walking options, car used	16	1.4%
Car and PT used	15	1.3%
PT used	13	1.1%
No modal options	12	1.0%
Cycling, PT and car used	11	1.0%
PT option, car used	11	1.0%
Cycling and walking options, PT and car used	11	1.0%
PT option, cycling and walking used	10	0.9%
Car option, walking and PT used	10	0.9%
PT and car options, cycling and walking used	9	0.8%
Car used	8	0.7%
PT option, no modal options used	8	0.7%
Cycling option, car used	6	0.5%
Cycling option, PT used	6	0.5%
PT option, cycling and car used	6	0.5%
Cycling, walking and car used	6	0.5%
Cycling option, walking and car used	5	0.4%
Cycling and PT options, walking used	5	0.4%
Walking used	5	0.4%
Cycling and PT used	4	0.3%
PT and car options, walking used	4	0.3%
Car option, PT used	3	0.3%
Walking and car used	3	0.3%
PT option, walking used	3	0.3%
Cycling and PT options, car used	3	0.3%
Cycling, walking and car options, PT used	3	0.3%
Cycling and walking options, PT used	3	0.3%
Cycling, walking and PT options, no modal options used	2	0.2%
Walking and cycling used	2	0.2%
Cycling and car used	2	0.2%
Cycling, PT and car options, walking used	2	0.2%
Cycling option, no modal options used	1	0.1%
Cycling and car options, no modal options used	1	0.1%
Walking and cycling options, no modal options used	1	0.1%
PT and car options, no modal options used	1	0.1%
Car option, no modal options used	1	0.1%
PT option, cycling used	1	0.1%
PT and car options, cycling used	1	0.1%
Cycling and PT options	1	0.1%
Walking option, cycling used	1	0.1%
Cycling, walking, PT and car options, no modal options used	1	0.1%
Total	1149	100.0

Note: PT—indicates public transport.

**Table 2:** Socio-demographic differences in modal choice processes.

Selection Process Description	Income % (*n*)		Gender % (*n*)		Household Status % (*n*)	
	Lower Income *	Higher Income (+Remainder of Respondents)	Men	Women	Not Cohabiting	Cohabiting
Walking and PT used	50% (41)	50% (41)	21% (17)	79% (65)	68% (56)	32% (26)
PT option, walking and car used	28% (9)	72% (23)	56% (18)	44% (14)	19% (6)	81% (26)
Walking, PT and car used	23% (24)	77% (80)	45% (47)	55% (57)	43% (45)	57% (59)
Cycling option, walking and PT used	42% (14)	58% (19)	6% (2)	94% (31)	61% (20)	39% (13)
Cycling, walking and PT options, car used	21% (6)	79% (22)	75% (21)	25% (7)	29% (8)	71% (20)
Cycling and PT options, walking and car used	17% (7)	83% (33)	73% (29)	27% (11)	10% (4)	90% (36)
Cycling option, walking, PT and car used	15% (16)	85% (88)	52% (54)	48% (50)	20% (21)	80% (83)
Cycling, walking and PT used	43% (27)	57% (35)	21% (13)	79% (49)	65% (40)	35% (22)
PT option, cycling, walking and car used	24% (20)	76% (62)	59% (48)	41% (34)	18% (15)	82% (67)
Cycling, walking, PT and car used	19% (63)	81% (268)	53% (174)	47% (157)	24% (79)	76% (252)
Total	28%	72%	46%	54%	33%	67%

Note: * A lower income refers to those with a monthly household income of up to 16,666 Swedish crowns (SEK).

**Table 3:** Reasons for choosing to use one or several modes over another or other options ^1^.

Code Description	Frequency of Mentions	Percentage
More suitable (or less trouble than other modes)	487	36.3%
More comfortable (e.g., having a place to sit)	310	23.1%
Faster/speed	86	6.4%
Health reasons/poor health/no energy	48	3.6%
Habit/not a conscious choice	45	3.4%
Less expensive/financial reasons	43	3.2%
Proximity	40	3.0%
Enjoyable (e.g., the experience. the views/less unenjoyable)	31	2.3%
No choice/not much choice/no alternatives	28	2.1%
Reliable (e.g., punctuality)	26	1.9%
These are the options available	24	1.8%
No need for other options/alternatives	21	1.6%
Better for the environment/environmental reasons	20	1.5%
Exercise/wants to move as much as possible	19	1.4%
More secure (e.g., lower risk of being exposed to crime)	19	1.4%
Safer (traffic/road safety)	19	1.4%
Flexible depending on the situation/use all options/ strategic use	18	1.3%
Distance/too far to walk	13	1.0%
Due to someone else’s needs or wants/ looking after someone else/someone else decides/ shared household resources/ minding dog/grandchildren	11	0.8%
Adjustment with age or health circumstances/no need to travel/too much difficulty travelling/’too old’ to travel	7	0.5%
Freedom (general)	6	0.5%
The car is a given/normalised	6	0.5%
Too much trouble with the car/parking	4	0.3%
Satisfied with option(s)/with freedom of choice	3	0.2%
Transporting heavy or large items/loads	3	0.2%
Flexibility/no timetable/no planning	2	0.2%
Public transport not possible/does not exist	2	0.2%
**Total**	**1341**	**100%**

Note: ^1^ Frequency of mentions, several reasons given by some respondents (*n* = 961), with some respondents choosing not to give a reason for their modal selection.

**Table 4:** Theme descriptions and the descriptions of codes included in each theme.

Theme Description	Descriptions of Codes Included
More comfortable (or previous combination of more suitable and more comfortable)	More suitable (or less trouble than other modes) More comfortable (e.g., having a place to sit)
Flexibility and versatility/habit	Habit/not a conscious choice Distance/too far to walk Transporting heavy or large items/loads Flexibility/no timetable/no planning The car is a given/normalized
Less expensive/financial reasons	Less expensive/financial reasons
Security and safety	More secure (e.g., lower risk of being exposed to crime) More secure and more comfortable Safer (traffic/road safety)
More suitable	More suitable
Extra benefits (e.g., freedom, enjoyment, exercise, environment)	Enjoyable (e.g., the experience. the views/less unenjoyable) Exercise/wants to move as much as possible Better for the environment/environmental reasons Enjoyable and better for the environment Freedom (general)
Reliability, punctuality and speed	Faster/speed Reliable (e.g., punctuality)
Expressing a limitation with options (e.g., less choice, adjusted expectations or trouble with car)	Public transport not possible/does not exist No choice/not much choice/no alternatives Adjustment with age or health circumstances/no need to travel/too much difficulty travelling/‘too old’ to travel Too much trouble with the car/parking These are the options available
Health reasons/poor health/no energy	Health reasons/poor health/no energy
Expressing satisfaction with options (e.g., use several options, no need for other options)	Flexible depending on the situation/use all options/ strategic use Satisfied with option(s)/freedom of choice No need for other options/alternatives
Decision dependent on someone else or the needs of others	Due to someone else’s needs or wants/ looking after someone else/someone else decides/ shared household resources/ minding dog/grandchildren

**Table 5:** Cross-tabulation of selection process groups of interest with themes of reasons for modal selection based on the first recorded reason (*n* = 395) *.

Selection Processes Examined Further	More Suitable % (*n*)	More Comfortable (or Previous Combination of More Suitable and More Comfortable) % (*n*)	Reliability, Punctuality and Speed % (*n*)	Flexibility and Versatility/Habit % (*n*)	Less Expensive/Financial Reasons % (*n*)	Expressing a Limitation with Options (e.g., Less Choice, Adjusted Expectations or Trouble with Car) % (*n*)	Expressing Satisfaction with Options (e.g., Use Several Options, No Need for Other Options) % (*n*)	Extra Benefits (e.g., Freedom, Enjoyment, Exercise, Environment) % (*n*)	Security or Safety % (*n*)	Health Reasons/Poor Health/No Energy % (*n*)	Decision Dependent on Someone Else or the Needs of Others % (*n*)	Total % (*n*)
PT option, car used	30.0% (3)	10.0% (1)	10.0% (1)	0.0% (0)	0.0% (0)	10.0% (1)	0.0% (0)	0.0% (0)	0.0% (0)	40.0% (4)	0.0% (0)	100.0% (10)
PT option, car and walking used	53.1% (17)	28.1% (9)	9.4% (3)	3.1% (1)	0.0% (0)	3.1% (1)	0.0% (0)	0.0% (0)	0.0% (0)	0.0% (0)	3.1% (1)	100.0% (32)
Car option. walking and PT used	70.0% (7)	0.0% (0)	0.0% (0)	10.0% (10)	0.0% (0)	0.0% (0)	10.0% (1)	0.0% (0)	0.0% (0)	0.0% (0)	10.0% (1)	100.0% (10)
Cycling option, PT and car used	64.7% (11)	0.0% (0)	5.9% (1)	0.0% (0)	5.9% (1)	0.0% (0)	0.0% (0)	0.0% (0)	0.0% (0)	23.5% (4)	0.0% (0)	100.0% (17)
Car option, cycling and PT used	62.5% (10)	0.0% (0)	6.3% (1)	0.0% (0)	6.3% (1)	0.0% (0)	18.8% (3)	0.0% (0)	6.3% (1)	0.0% (0)	0.0% (0)	100.0% (16)
Cycling and walking options, car used	43.8% (7)	18.8% (3)	6.3% (1)	12.5% (2)	6.3% (1)	12.5% (2)	0.0% (0)	0.0% (0)	0.0% (0)	0.0% (0)	0.0% (0)	100.0% (16)
Cycling option, walking and PT used	45.5% (15)	6.1% (2)	6.1% (2)	12.1% (4)	3.0% (1)	18.2% (3)	0.0% (0)	0.0% (0)	6.1% (2)	3.0% (1)	0.0% (0)	100.0% (33)
Cycling, walking and PT options, car used	42.9% (12)	25.0% (7)	7.1% (2)	10.7% (3)	0.0% (0)	3.6% (1)	0.0% (0)	7.1% (2)	0.0% (0)	3.6% (1)	0.0% (0)	100.0% (28)
Cycling and PT options, walking and car used	45.0% (18)	35.0% (14)	2.5% (1)	0.0% (0)	0.0% (0)	0.0% (0)	7.5% (3)	7.5% (3)	0.0% (0)	2.5% (1)	0.0% (0)	100.0% (40)
Cycling option, walking, PT and car used	55.3% (57)	21.4% (22)	2.9% (3)	2.9% (3)	2.9% (3)	6.8% (7)	3.9% (4)	1.0% (1)	2.9% (3)	0.0% (0)	0.0% (0)	100.0% (103)
PT and car options, walking and cycling used	25.0% (2)	37.5% (3)	0.0% (0)	0.0% (0)	0.0% (0)	0.0% (0)	37.5% (3)	0.0% (0)	0.0% (0)	0.0% (0)	0.0% (0)	100.0% (8)
PT option, cycling, walking and car used	47.6% (39)	30.5% (25)	6.1% (5)	3.7% (3)	1.2% (1)	1.2% (1)	2.4% (2)	4.9% (4)	0.0% (0)	1.2% (1)	1.2% (1)	100.0% (82)
Total	50.1% (198)	21.8% (86)	5.1% (20)	4.3% (17)	2.0% (8)	4.8% (19)	4.1% (16)	2.5% (10)	1.5% (6)	3.0% (12)	0.8% (3)	100.0% (395)

* Please note that this table is simply intended to give an indication of how respondents answered and some of the percentages represent very small numbers. PT—indicates public transport.
